# The value of the reflective discussion in decision-making using multi-criteria decision analysis (MCDA): an example of determining the value contribution of tabelecleucel for the treatment of the Epstein Barr virus-positive post-transplant lymphoproliferative disease (EBV^+^ PTLD)

**DOI:** 10.1186/s13023-024-03324-5

**Published:** 2024-08-23

**Authors:** Xavier Badia, Miguel Ángel Calleja, Vicente Escudero-Vilaplana, Antonio Pérez-Martínez, José Luis Piñana, José Luis Poveda, Joan-Antoni Vallès

**Affiliations:** 1 Omakase Consulting S.L., Barcelona y Madrid, Spain; 2 Hospital Universitario Virgen Macarena, Seville, Spain; 3 Hospital General Universitario Gregorio Marañon, Madrid, Spain; 4 University Hospital La Paz, Madrid, Spain; 5 University Clinical Hospital of Valencia, Valencia, Spain; 6 La Fe University and Polytechnic Hospital, Valencia, Spain; 7 Catalan Institute of Health, Catalonia, Spain

**Keywords:** Reflective discussion, Decision-making, MCDA, EBV + PTLD

## Abstract

**Background:**

The aim of this study was to assess the contribution of the reflective multidisciplinary discussion in determining the value contribution of innovative drugs through the multi-criteria decision analysis (MCDA). This methodology considers all relevant criteria for healthcare decision-making in a global, transparent, and systematic manner and from the perspective of relevant stakeholders. The determination of value contribution of tabelecleucel for the treatment of Epstein-Barr virus-positive post-transplant lymphoproliferative disease (EBV^+^ PTLD) compared to salvage therapy was used as an example.

**Results:**

Tabelecleucel obtained a value contribution score of 0.63 and increased to 0.75 after the reflective discussion. EBV^+^ PTLD was considered a life-threatening disease (5.0 ± 0.0), with a significant unmet need for an approved treatment (5.0 ± 0.0). Tabelecleucel was perceived as bringing improvements in terms of efficacy (4.2 ± 0.8) and safety (3.8 ± 0.8) compared to the salvage therapy. Most experts considered that the high efficacy and safety results could represent an improvement in the quality of life of patients (2.3 ± 1.2) along with savings in medical costs (2.3 ± 2.0) and non-medical costs (2.7 ± 1.6) compared to the salvage therapy. However, others emphasized the need of more evidence to confirm these improvements and savings over time. Tabelecleucel was regarded as potentially modifying the clinical course of the disease (4.3 ± 0.8) and supported by high-quality evidence (3.2 ± 0.4). All contextual criteria were valued highly positively for tabelecleucel. "Safety/Tolerability" and "Other medical costs" were the criteria that experienced the highest change in the re-test conducted after the reflective discussion. The reflective discussion allowed resolving doubts or misinterpretations of the experts, so the re-test obtained more accurate and consistent results of the value contribution of tabelecleucel.

**Conclusions:**

The study shows that the MCDA methodology is a useful tool for decision-making on innovative treatments for the management of rare diseases. It also highlights the importance of reflective multidisciplinary discussion for its ability to resolve doubts or misinterpretations of experts, subsequently allowing to obtain more consistent and reliable results on the value contribution of the drug, being potentially more positive.

## Background

Healthcare reimbursement decisions for drugs indicated to treat rare diseases are challenging as the strength of evidence for these drugs is often limited by the inherent natural history of the disease. Consequently, the appraisal of the value and most appropriate positioning within healthcare systems of an orphan drug should be holistic, requiring a broader perspective, and not limited to the traditional criteria of efficacy, safety and cost.

Reflective Multi-Criteria Decision Analysis (MCDA) offers a methodology that allows the determination of what represents value in each therapeutic indication considering all relevant criteria for healthcare decision-making in a holistic, transparent, and systematic manner and from the perspective of relevant stakeholders [1]. Furthermore, it enables a comprehensive and multidisciplinary analysis of its global value by considering the reflections of all stakeholders involved in decision-making [1–4].

The most important part of the MCDA methodology is the reflective discussion where the stakeholders collectively share their perception of the value contribution of the drug in detail considering all relevant decision-making criteria.

Post-transplant lymphoproliferative disease (PTLD) is a rare and potentially deadly haematological malignancy that can occur after an allogeneic haematopoietic cell transplant (HCT) or solid organ transplant (SOT). PTLD is frequently associated with Epstein-Barr virus (EBV), an oncogenic virus presents in 95% of the adult population [5]. EBV can cause the uncontrolled growth and proliferation of infected B lymphocytes in patients under immunosuppressed conditions, as such induced in patients after transplantation, generating Epstein-Barr virus positive post-transplant lymphoproliferative disease (EBV^+^ PTLD)[6]. EBV is the most common cause of PTLD following HCT or SOT transplantation, accounting for nearly all cases of HCT and approximately 50% of SOT [7, 8].

EBV^+^ PTLD is an ultra-rare disease with an estimated annual incidence of less than 1 in one million [9–11]. EBV^+^ PTLD affects transplant recipients of all ages, including children, resulting in a patient population that is on average 25–30 years younger at diagnosis compared to patients with other lymphomas [9, 10].

EBV^+^ PTLD is a life-threatening disease with a short survival period. Patients with EBV^+^ PTLD who fail initial therapy, experience a worsened clinical burden with complications and poor outcomes, high mortality rates, with median overall survival between 0.7 and 1.7 months for patients with HCT [11, 12] and between 3.3 and 4.1 months for patients with SOT [13, 14]. Once these treatments are exhausted, there are currently no drug alternatives apart from supportive care, such as salvage chemotherapy [8].

Tabelecleucel is the first and only therapeutic option approved by the European Commission (EC) for EBV^+^ PTLD patients after failing standard of care therapy and is indicated as monotherapy for treatment of adult and paediatric patients 2 years of age and older with relapsed or refractory (R/R) EBV + PTLD who have received at least one prior therapy. For solid organ transplant patients, prior therapy includes chemotherapy unless chemotherapy is inappropriate [15, 16].

The aim of this study was to determine the contribution of the reflective multidisciplinary discussion in determining the value contribution of innovative drugs through the MCDA criteria used for decision-making. The assessment of tabelecleucel for the treatment of R/R EBV^+^ PTLD compared to salvage therapy was used as an example.

## Methods

### Study design

The present study analysed the value contribution of tabelecleucel compared to the salvage therapy used in clinical practice through MCDA. The MCDA methodology used in this study is based on the EVIDEM framework that was adapted to Spain [17] and then to the evaluation of orphan drugs [18]. This framework has been used in several studies [19–22] as it has been already considered by evaluators and decision-makers as a complete and useful tool, feasible to be used for orphan drug evaluation and decision-making at national level [19, 23, 24]. The EVIDEM reflective framework stimulates structured reflection from stakeholders through a set of quantitative and qualitative criteria that integrate the ethical underpinning of decision-making [1]. The used framework is composed of 9 quantitative (two disease-related and seven drug-related) and 4 qualitative or contextual criteria focused on the consideration of the context surrounding decision-making [18, 25]. The rare disease framework used in the present study is shown in Table 1. Quantitative criteria are scored on a scale of 0 to 5 for non-comparative criteria (“Disease Severity”; “Unmet Needs”; “Therapeutic Impact”; “Quality of Evidence or Level of Evidence and Recommendation Grade”), and -5 to 5 for comparative criteria (“Efficacy/Effectiveness”; “Safety/Tolerability”; “Patient-Reported Outcomes”; “Other Medical Costs”; “Non-Medical/Indirect Costs”), with 5 being the most favourable score in both cases. The evaluation of contextual criteria was performed on a qualitative scale, defined as positive, neutral, or negative impact.

**Table 1:** Quantitative and qualitative criteria of the EVIDEM framework adapted and validated for orphan drugs [25]

Domain: Impact on the disease Disease severity Unmet needs	Domain: Normative context Population access priorities Common goal and specific interests
Domain: Comparative outcomes of the treatment Comparative Efficacy/effectiveness Comparative Safety/tolerability Comparative Patient-reported outcomes (PROs)	Domain: Feasibility System capacity and appropriate use of the intervention Mandate and scope of the healthcare system
Domain: Type of benefit of the treatment Therapeutic impact	
Domain: Economic consequences of the treatment Other medical costs Non-medical/indirect costs	
Domain: Knowledge about the treatment Quality of evidence or level of evidence and recommendation grade	

### Comparator

Due to the absence of an approved treatment for patients with R/R EBV^+^ PTLD, supportive care, including salvage chemotherapy, was considered as a comparator to tabelecleucel for the study. However, it does not constitute an evidence-based alternative, due to a lack of standardization of regimens [8, 26, 26, 27].

The steps followed for the study are presented below:

### Literature review

A systematic literature review was conducted to collect relevant information about the disease and its management in Spain, then the data were compiled into each criterion of the evidence matrix. The literature review was carried out between May and June 2022 according to a protocol including the criteria of the adapted MCDA framework for the evaluation of orphan drugs in Spain [25, 28].

All articles identified through the search were screened by title and abstract. Articles not responding to the search objective, or not meeting eligibility criteria were excluded (Table 2). Moreover, duplicated articles, articles written in a language other than Spanish or English, or related to animal studies, were also excluded. A full-text assessment was performed with those remaining.

**Table 2:** Inclusion and exclusion criteria for literature review

Articles related to the burden of disease, patient journey, disease management and unmet needs associated with EBV + PTLD in Spain Diagnosis and treatment guidelines, clinical recommendations, treatment algorithms and consensus Articles published in peer-reviewed journals, systematic reviews, meta-analyses, articles published by patient associations, or information published by HTA agencies Relevant information from clinical trial registries Available in Spanish and English	Duplicated publications Publications related to animal studies Studies based on non-pharmacological disease management (e.g., smoking cessation, exercise) Studies mentioning EBV + PTLD, but focusing on other diseases

EBV: Epstein Barr Virus; HTA: Health Technology Assessment; PTLD: Post-Transplant Lymphoproliferative Disease

Published evidence was searched using the biomedical databases PubMed [29], and MEDES [30], with no time span limit applied to the publications. Additionally, the research was complemented by using grey literature sources, such as Google Scholar, and by consulting the websites of relevant scientific societies and patient organizations.

### Criteria weighting

The weights of the relative importance of quantitative criteria were obtained from a previous study conducted at the national level, where 98 national and regional stakeholders from the healthcare sector worked in drug evaluation committees in Spain [31].

### Expert panel

The study was conducted with a multidisciplinary panel of six professionals with significant expertise in the management and treatment of patients with EBV^+^ PTLD and in decision-making: 2 haematologists head of leading haematological units (paediatric and adult), 2 heads of hospital pharmacy, 1 hospital pharmacy specialised in haematology, and 1 pharmacologist involved in regional appraisal of innovative drug therapy. One of the haematologists had previous experience with tabelecleucel.

All participants received a 1-h online training (held in July 2023) on the reflective MCDA methodology and were introduced to the evidence matrix. Subsequently, the expert panel remotely scored each criterion in the evidence matrix including reflection on the rationale for the scoring. Due to the rarity of the disease, the information presented in some criteria of the evidence matrix may be limited. In these cases, we used the advice agreed with the Orphar-SEFH group that, when criteria do not have available information, these could be scored based on the interpretation of the available evidence of efficacy, safety and quality of life.

Then, a quantitative analysis of the scores was conducted, and the comments and reflections behind experts’ scores were collected in a qualitative manner.

### Reflective discussion and re-scoring

After the expert panel scored the matrix and scores were analysed, a reflective discussion was held to present the results and discuss them collectively (also held in July 2023). Then, after the reflective discussion, participants were asked to re-score the same evidence matrix during the following week of the meeting considering the reflective discussion held during the meeting. Participants were allowed to change their ratings explaining the reasons behind the potential change. The re-test aimed to assess the impact of the reflective multidisciplinary discussion on the score and to increase the consistency and reliability of the study result $(22)$.

### Data analysis

The scores for each criterion rated by the expert panel were individually collected from each participant and consolidated into a unified database. Data analysis was run in Microsoft Excel. Mean $\left({S}_{X}\right)$, standard deviation $\left(SD\right)$, median, and range of scores (minimum and maximum) were calculated for the 9 quantitative criteria.

The value contribution $\left(VC\right)$ for each of the 9 quantitative criteria were determined by using the normalised scores $\left(Se=score/5\right)$ multiplied by the value of each criterion relative weight $\left({VC}_{W}\right) \left(\sum {VC}_{W}=1\right)$: $\left(VC={{S}_{e}*VC}_{W}\right)$. The overall value score $\left(OVC\right)$, a value that goes from 0 to 1, is the sum of all quantitative criteria value contributions $\left(OVC=\sum VC\right) (1)$. Comments and reflections behind experts´ scores were analysed and recorded in a qualitative manner.

For contextual criteria, grades were transformed to a numerical scale as + 1 for positive, 0 for neutral, and -1 for negative opinion. The results are shown as the percentage of the experts' ratings on the drug's impact according to each contextual criterion definition.

For the re-test performed during the following week after the reflective discussion, the scores for each criterion rated by the expert were analysed in the same way as presented above. The difference in the means obtained for each criterion between the first score and the second score was calculated, and the reason and rationale raised during the reflective discussion were described.

Due to the small sample size and the qualitative nature of the study, we only used descriptive statistics.

## Results

### Literature review

**Fig. 1:**
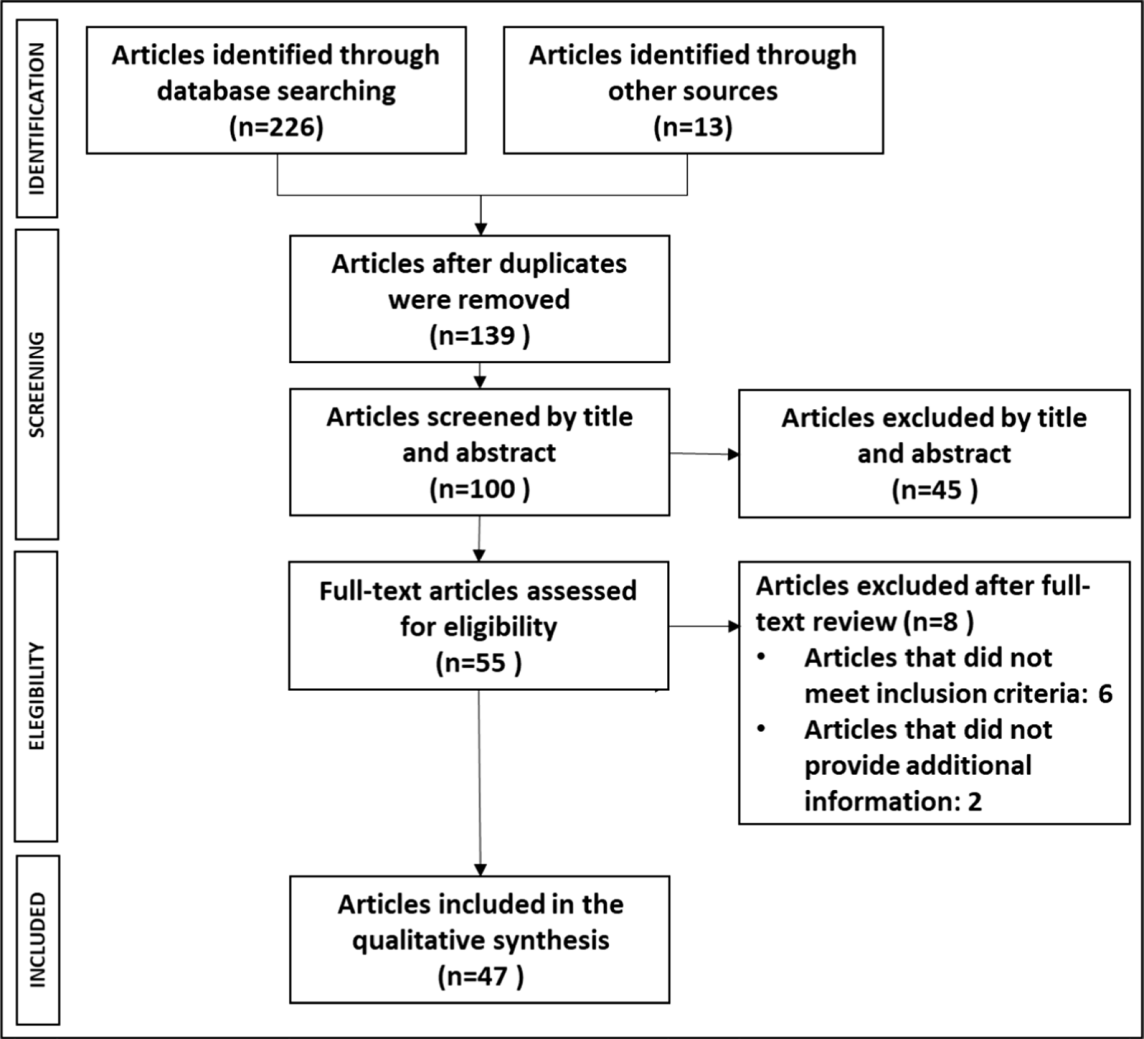
PRISMA flowchart of the literature review results

In total, 239 articles were identified, 226 obtained from biomedical databases and 13 from grey literature sources. After compiling these publications into a single database, 139 (58.2%) duplicate articles were eliminated. The remaining 100 articles were screened by title and abstract and 45 (45%) were excluded as they did not meet the inclusion criteria for this literature review. An additional eight articles were discarded following a full-text review for not meeting the inclusion criteria (6 articles) or for offering no new information compared to other identified publications (2 articles). Finally, 47 articles were used to compile the evidence matrix. The PRISMA diagram with the results of the literature review is shown in Fig. 1.

### Quantitative criteria

**Fig. 2:**
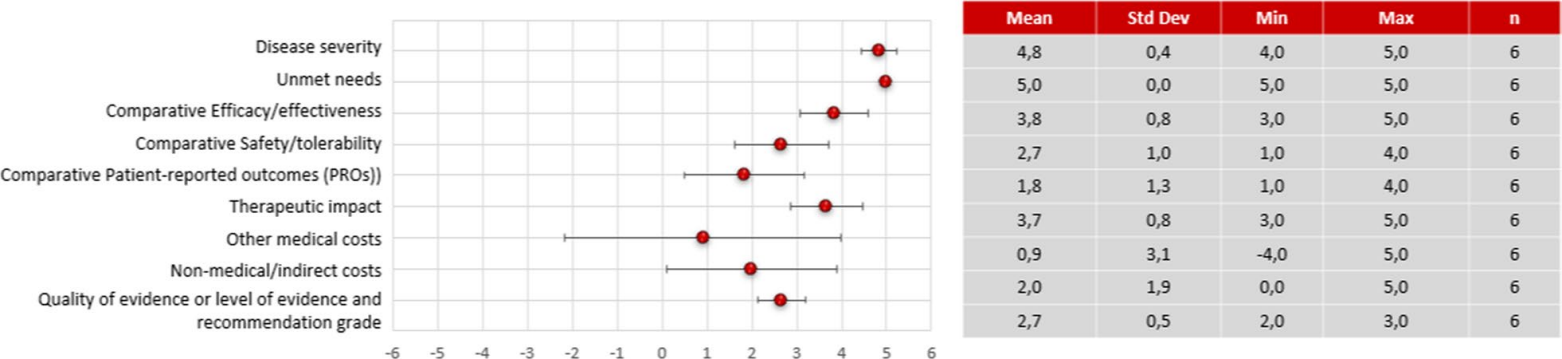
Quantitative criteria score results of tabelecleucel compared to the salvage therapy before reflective discussion. Dots correspond to the mean of the scores given by the 6 participants, and the bars show the standard deviation. A constructed, cardinal scoring scale was used, ranging from 0 to 5 for non-comparative and from −5 to 5 for comparative criteria, respectively

The results of the expert panel's scoring on the quantitative criteria of the evidence matrix are shown in Fig. 2.

### Disease severity

EBV^+^ PTLD was rated by all experts as a very severe disease (4.8 [0.4]) because it was associated with a high mortality rate and extremely short survival period in transplant recipients who are R/R EBV^+^ PTLD after first-line treatment.

### Unmet needs

All participants agreed that there is an unmet medical need in the management of R/R EBV^+^ PTLD (5 [0.0]). They consider there is a need for an approved treatment that is effective in terms of survival and with minimal toxic effects for EBV + PTLD patients refractory or relapsed to initial therapy, thus decreasing the clinical burden of disease.

### Comparative efficacy/effectiveness

The expert panel rated efficacy/effectiveness of tabelecleucel (3.8 ± 0.8) in patients with R/R EBV^+^ PTLD following HCT and SOT significantly better compared to the salvage therapy but there was a slight variation in their reflections. Some participants considered that tabelecleucel showed a good overall survival result compared to the salvage therapy, but these could be improved as there is a percentage of patients who partially respond, especially SOT patients. During the discussion, haematologists highlighted that the most appropriate comparator for tabelecleucel would be the supportive therapy (salvage chemotherapy, despite not being an evidence-based alternative due to the lack of standardisation of regimens), as patients are refractory to first-line treatment, and some participants realised that they had not taken this into account when scoring the criteria. Therefore, they commented that this would be a reason to increase the previous score assigned for this criterion. Other participants pointed out some methodological limitations in the pivotal study, such as the small number of patients and the absence of a comparator arm, although these were considered inherent to rare diseases. Furthermore, the better results of tabelecleucel in HCT patients vs. SOT patients were mentioned, but one participant highlighted that the withdrawal of immunosuppressants in EBV^+^ PTLD patients could be the reason.

### Comparative safety/tolerability

The safety and tolerability profile of tabelecleucel was rated by the expert panel as superior compared to salvage therapy (2.7 ± 1.0) but there was variability in their score. All participants of the expert panel considered the safety profile of tabelecleucel to be favourable and manageable compared to salvage therapy. However, one participant highlighted that uncertainty remains due to the small sample size used in the studies to assess the safety profile and the limited experience in managing adverse events related to a new treatment. During the discussion, it was noted that some participants scored lower due to misinterpretation of the information. They did not consider salvage therapy as the most representative comparator for tabelecleucel but instead considered rituximab, and they associated tumour exacerbation and pneumonitis as treatment-related adverse effects.

### Comparative patient-reported outcomes (PROs)

Tabelecleucel was rated to be superior in terms of PROs compared to salvage therapy (1.8 ± 1.3) but there was a high variability in the scores. Most experts considered that the demonstrated efficacy and adequate safety profile of tabelecleucel could represent an improvement in patients' quality of life despite the lack of available data on PROs. However, other participants agreed that the limited evidence published to date is insufficient to conclude the effect of tabelecleucel on patients' quality of life [15, 32].

### Therapeutic impact

The therapeutic impact of tabelecleucel in the treatment of EBV^+^ PTLD was also scored positively across all participants (3.7 ± 0.8), as it modifies the clinical course of the disease. In addition, experts highlighted that there is experience in clinical practice with similar T-cell therapies that have demonstrated a good long-term efficacy profile in the control of EBV-infected infections and tumour cells. Some participants had some uncertainties regarding the importance of HLA compatibility between patient and donor, the most appropriate dose and the frequency of infusions required according to the patient's profile; however, during the discussion, experts agreed that these limitations would be solved with future experience of the product in clinical practice.

### Other medical costs

There was observed high variability in the scores for the medical cost criterion (excluding pharmacological cost) associated with tabelecleucel assigned by the experts (0.9 ± 3.1). Some participants considered that tabelecleucel could lead to medium-term savings for the system, as it is a more effective, safer, and more convenient therapy than the salvage therapy, easy to use due to being an allogeneic treatment, resulting in lower costs for hospital care and palliative care admissions. In addition, patients who respond would avoid organ rejection, which is an important ethical criterion to consider. However, two of the six participants pointed out that the percentage of non-responders is higher with chemotherapy versus tabelecleucel, so, as it is a very aggressive and non-chronic disease, a higher percentage of chemotherapy patients do not generate medical costs as they die in a very short period of time. During the reflective discussion it emerged that, for a correct scoring of the criterion, the medical costs generated by a living patient, i.e. the costs per unit of benefit, have to be considered.

### Non-medical/indirect costs

Tabelecleucel was perceived as a therapeutic option that can produce savings in non-medical costs compared to the salvage therapy (2.0 ± 1.9). Its higher efficacy would decrease the burden of disease to the family and caregivers, and would potentially increase patient productivity in the future. However, uncertainty was also expressed due to the fast disease progression and the lack of clear evidence of this potential savings with tabelecleucel in the medium to long term.

### Quality of the evidence

The quality of the evidence supporting tabelecleucel was rated as moderate by all participants (2.7 ± 0.5). They pointed out certain limitations such as the absence of a control group, and a small sample size. However, they also recognised that these limitations are inherent to the rarity and life-threatening of the disease.

### Value contribution of tabelecleucel versus salvage therapy

**Fig. 3:**
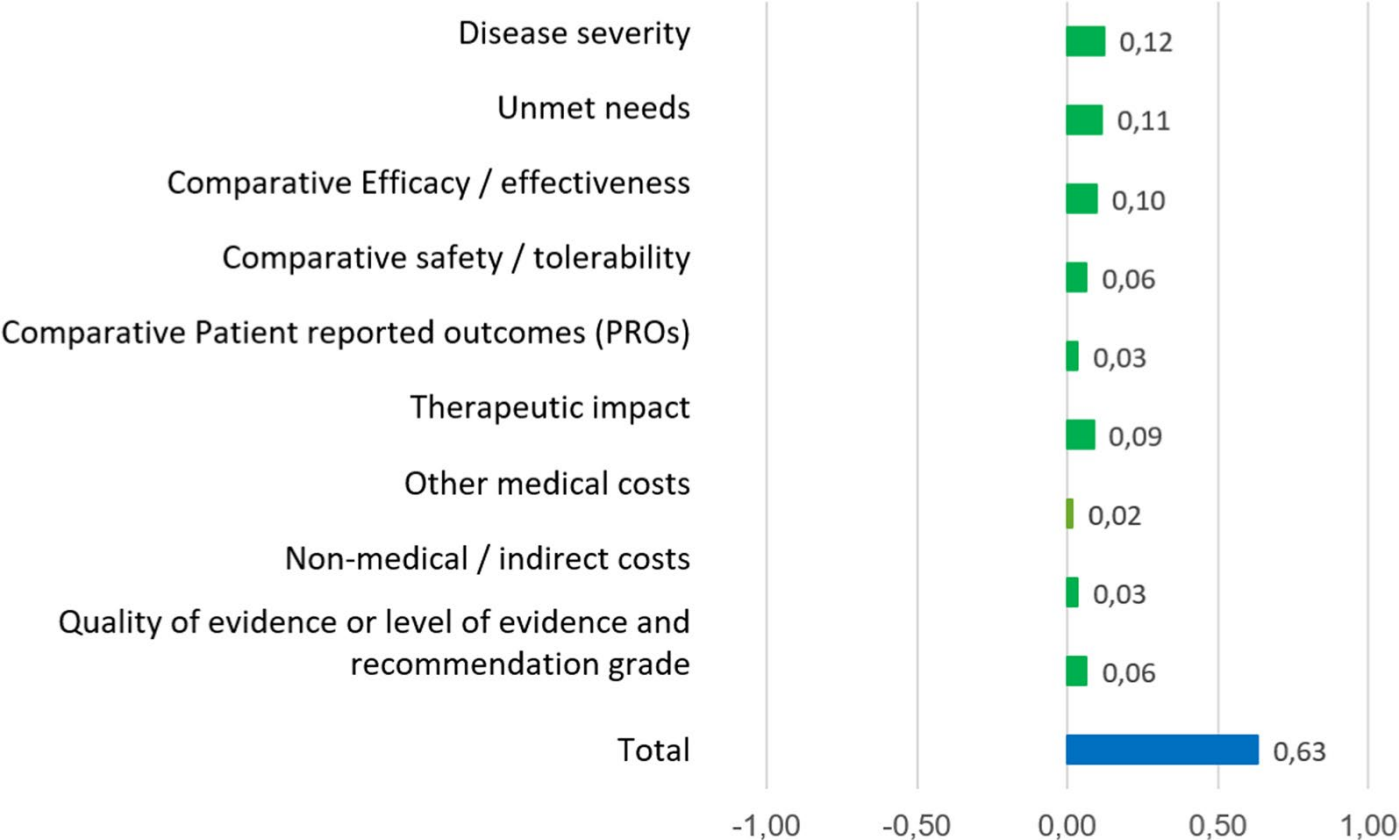
Test results of the standardized global value contribution of tabelecleucel vs salvage therapy

The value contribution for tabelecleucel vs. salvage therapy obtained is shown in Fig. 3. Tabelecleucel’s global value contribution for the treatment of R/R EBV^+^ PTLD was perceived to be superior compared to salvage therapy (score: 0.63). The greatest value contribution of tabelecleucel was observed in the criteria of “Disease Severity”, “Unmet Needs”, “Efficacy/Effectiveness”, and “Therapeutic Impact”.

### Contextual criteria

**Fig. 4:**
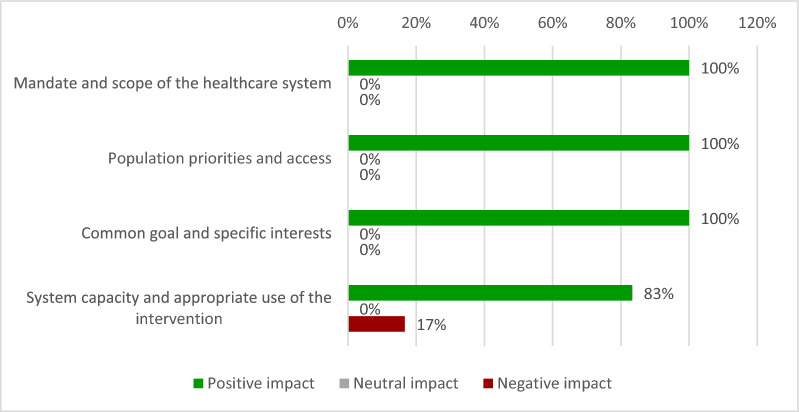
Results of the qualitative criteria score of tabelecleucel

Assessment of the qualitative criteria from the adapted MCDA framework was positive for all criteria (Fig. 4). Participants scored the qualitative criteria in terms of positive, neutral, or negative impact of tabelecleucel for each criterion.

All experts (100%) agreed that the use of tabelecleucel for the treatment of EBV^+^ PTLD would be aligned with the priorities of the National Health System (NHS) because it was considered that, as a rare disease with no available alternatives, tabelecleucel targets a patient population with high unmet needs. All experts (100%) agreed that the introduction of tabelecleucel for the treatment of patients with EBV^+^ PTLD in the NHS has a positive impact on the specific interests of this patient population, covering an unmet need.

All experts (100%) considered that tabelecleucel would be aligned with the objective and specific interests of the Spanish NHS and involved scientific societies, patient associations, and clinical practice guidelines.

Regarding the system capacity and appropriate use of the intervention most experts (83%) agreed that the NHS has the capability and infrastructure required to ensure the proper use of tabelecleucel in treating EBV^+^ PTLD. The experts stated that many centres have CAR-T therapies that require more labour-intensive infrastructure than tabelecleucel, although they also advised that the treatment should only be accessible to hospitals with experience in HCT and SOT. However, one hospital pharmacist considered that the inclusion of tabelecleucel might require a higher consumption of resources (storage, coordination with the Autonomous Community, adaptation of some pharmacy services, coordination between circuits…). On the other hand, a hospital pharmacist emphasised the problems of internal management with data recording and invoice matching for this type of non-single-dose drug.

### Test–retest after reflective discussion

**Fig. 5:**
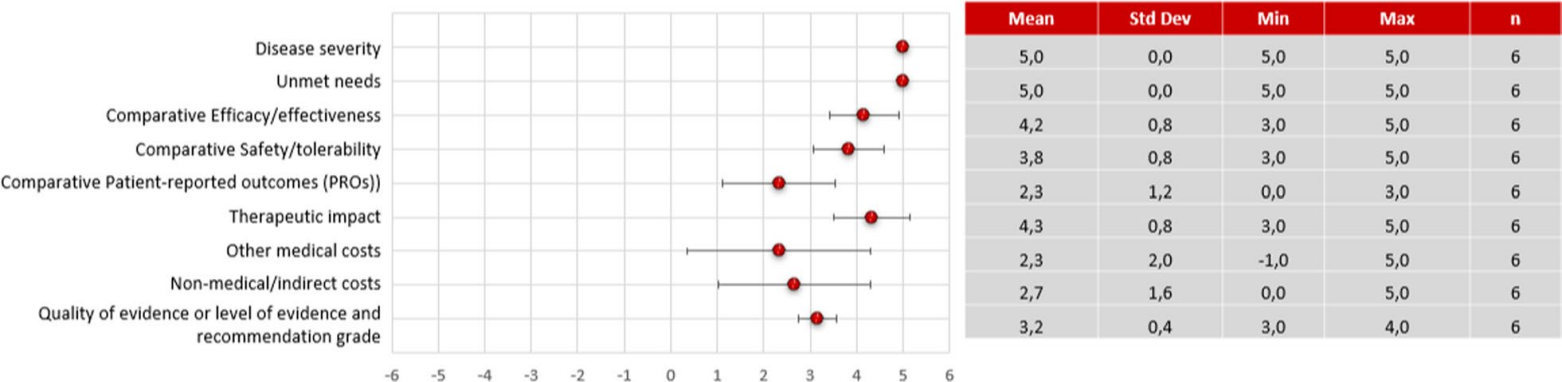
Quantitative criteria score results of tabelecleucel compared to the salvage therapy after reflective discussion. Dots of the figure correspond to the mean of the scores given by the 6 participants, and the bars show the standard deviation. A constructed, cardinal scoring scale was used, ranging from 0 to 5 for non-comparative and from −5 to 5 for comparative criteria, respectively

Ratings assigned by expert panel to quantitative criteria after the reflective discussion are shown in Fig. 5 and the differences in scores between the test and re-test are shown in Table 3. The main reasons for change are described below only in those criteria that scores changed:

**Table 3:** Results obtained from the test and re-test scores of the quantitative criteria and its difference

Quantitative criteria	Test score			Re-test score			Test and re-test difference
	Mean	Std Dev	Min–Max	Mean	Std Dev	Min–Max	
Disease severity	4.8	0.4	4.0–5.0	5.0	0.0	5.0–5.0	+ 0.2
Unmet needs	5.0	0.0	5.0–5.0	5.0	0.0	5.0–5.0	0.0
Comparative efficacy/effectiveness	3.8	0.8	3.0–5.0	4.2	0.8	3.0–5.0	+ 0.3
Comparative safety/tolerability	2.7	1.0	1.0–4.0	3.8	0.8	3.0–5.0	+ 1.2
PROs	1.8	1.3	1.0–4.0	2.3	1.2	0.0–3.0	+ 0.5
Therapeutic impact	3.7	0.8	3.0–5.0	4.3	0.8	3.0–5.0	+ 0.7
Other medical costs	0.9	3.1	−4.0–5.0	2.3	2.0	−1.0–5.0	+ 1.4
Non-medical/indirect costs	2.0	1.9	0.0–5.0	2.7	1.6	0.0–5.0	+ 0.7
Quality of evidence or level of evidence and recommendation grade	2.7	0.5	2.0–3.0	3.2	0.4	3.0–4.0	+ 0.5

### Disease severity

The mean rating increased from 4.8 to 5.0 (5.0 ± 0.0) as one participant increased the score. During the discussion, the severity of the EBV^+^ PTLD, considered a very acute and potentially life-threatening disease with no current possibility to withdraw immunosuppression, was strongly emphasised.

### Comparative efficacy/effectiveness

The mean score increased from 3.8 to 4.2 (4.2 ± 0.8) as two participants changed the score. During the discussion, it was clarified by haematologists that the most appropriate comparator for tabelecleucel would be the supportive therapy (salvage chemotherapy), as patients are already refractory to first-line treatment and there are no medicinal alternatives, and therefore they considered that a higher efficacy score could be assigned to tabelecleucel.

### Comparative safety/tolerability

The mean score raised from 2.7 to 3.8 (3.8 ± 0.8) as five participants increased their score after reflective discussion. Main rationales were the consideration of salvage chemotherapy as the most representative comparators for tabelecleucel, and the association of some serious adverse events, such as tumour exacerbation and pneumonitis, with the immunosuppression status rather than tabelecleucel. Therefore, after resolving these concerns, most experts decided to increase the score considering tabelecleucel as a safe drug with manageable adverse effects. Additionally, they emphasized that salvage therapy such as salvage chemotherapy are potentially more toxic.

### Comparative patient-reported outcomes (PROs)

The mean score increased from 1.8 to 2.3 (2.3 ± 1.2) after the reflective discussion although there was still some variability in the score, with three experts increasing the score and two decreasing it. Participants that increased the score considered that the good efficacy and safety results of tabelecleucel could represent an improvement in the quality of life of patients compared to the salvage therapy. However, other participants expressed that data on quality of life is needed to provide a more accurate assessment of this criteria.

### Therapeutic impact

The mean score increased from 3.7 to 4.3 (4.3 ± 0.8) as four participants increased the score. These participants increased their score as, during the discussion, the potential ability of tabelecleucel to modify the clinical course of R/R EBV^+^ PTLD was emphasised and, despite the uncertainty about some aspects of tabelecleucel, experts considered that these could be solved with future experience of the product in clinical practice.

### Other medical costs

The mean score increased from 0.9 to 2.3 (2.3 ± 2.0) after the reflective discussion although there was still some variability in the score, with three experts increasing the score and two decreasing it. Participants who increased their score considered that the higher efficacy and safety of tabelecleucel compared to salvage therapy could result in lower hospital care costs and palliative care admissions. In addition, they also highlighted the significant savings to the system of protecting the graft explant with the use of tabelecleucel. However, two participants who slightly decreased their score highlighted that more evidence is needed to adequately assess its impact on other medical costs.

### Non-medical/indirect costs

The mean score increased from 2.0 to 2.7 (2.7 ± 1.6) as two participants increased the score. After the reflective discussion, most participants considered that the higher efficacy and lower toxicity of tabelecleucel compared to the salvage therapy could result in more work productivity, lower social and familiar/caregivers’ costs, among others. However, some experts still believed the need for data to quantify these savings with tabelecleucel.

### Quality of the evidence

The mean score raised from 2.7 to 3.2 (3.2 ± 0.4) as two participants slightly increased their score. After the discussion, more experts considered the quality of the study design and the relevance of the data to be sufficiently robust and adequate in the current context despite the limitations inherent to severe and rare diseases.

### Standardized global value contribution of tabelecleucel after reflective discussion

**Fig. 6:**
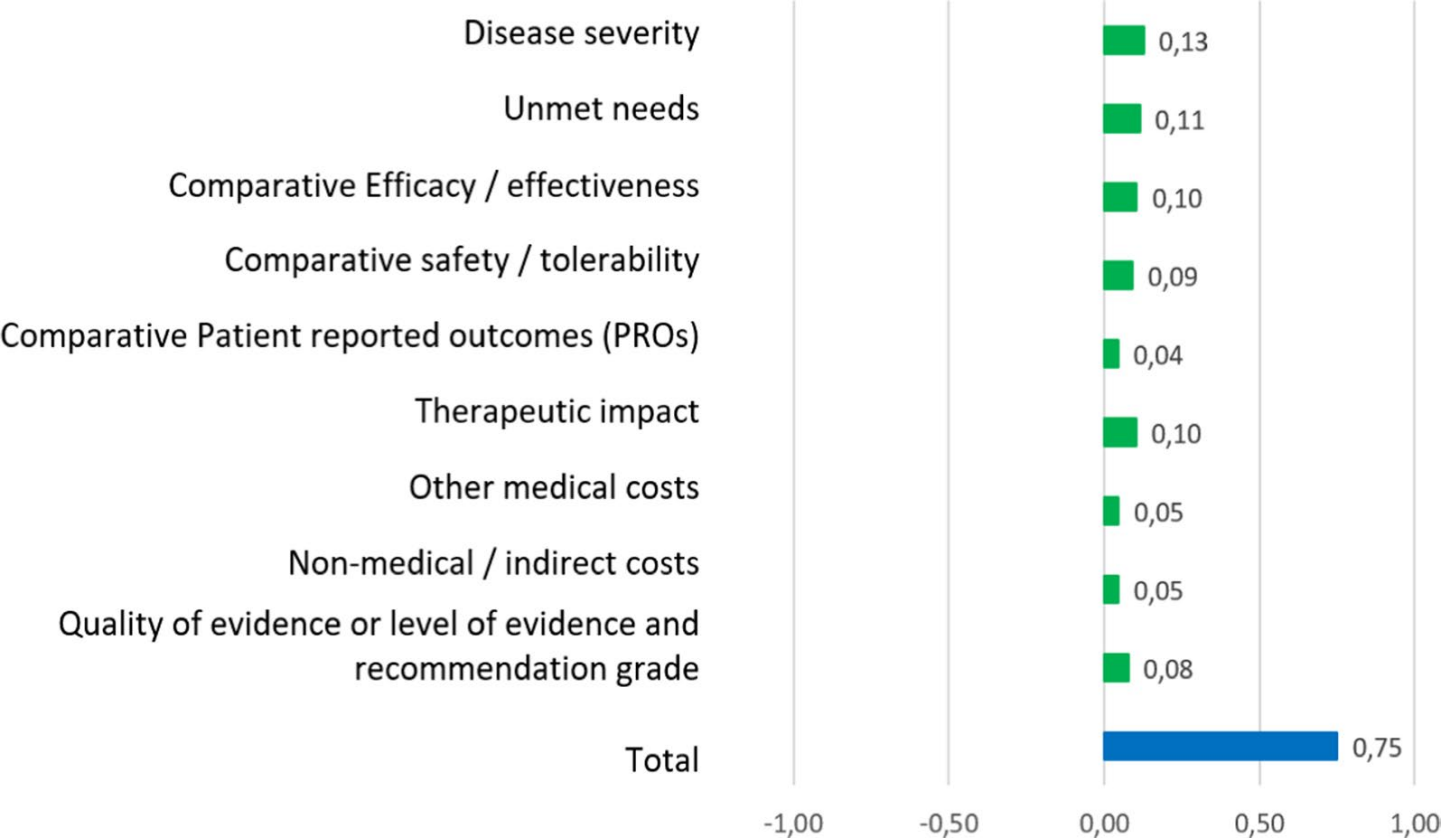
Re-test results of the standardized global value contribution of tabelecleucel vs salvage therapy

The overall value contribution of tabelecleucel vs. the salvage therapy obtained from the re-test increased from 0.63 to 0.75 (Fig. 6). An increase of 0.03 in the “Safety/Tolerability” and “Other medical costs” criteria, an increase of 0.02 in the “Non-medical/Indirect Cost” and “Quality of Evidence or Level of Evidence and Recommendation Grade” criteria, and an increase of 0.01 in the “Disease Severity”, “PRO” and “Therapeutic Impact” criteria were observed.

## Discussion

The aim of this study was to explore the contribution of reflective multidisciplinary discussion in decision-making, using as an example the study of determining the value of tabelecleucel for the treatment of EBV^+^ PTLD compared to the salvage therapy through MCDA.

The results obtained in this study suggest that tabelecleucel provides significant value for the treatment of R/R EBV^+^ PTLD disease compared to the salvage therapy. In this regard, tabelecleucel obtained an overall score of 0.63 and, after the reflective discussion, the value contribution increased to 0.75. The top three criteria contributing the most to the value of tabelecleucel were: “Disease Severity”, “Unmet Needs”, and “Efficacy/Effectiveness”; and the criteria that contributed the least were “Patient-Reported Outcomes (PROs)”, “Other costs” and “Non-medical/Indirect Costs”. The criteria experienced the highest change after the reflective discussion, increasing the score, were “Safety/Tolerability”, “Other medical costs”, “Non-medical/Indirect Cost”, and “Quality of Evidence”.

It has been found that the reflective discussion allows for the resolution of certain doubts or data that have been misinterpreted by the participants and shows that a different point of view is relevant to change the assessment. Therefore, in the re-test, less variability and better scores for tabelecleucel were shown in some criteria. This aspect underlines the positive impact that a reflective discussion among participants has on the rationale and interpretation of data, which shows whether re-testing should be done routinely in the healthcare context.

EBV^+^ PTLD was rated as a rare and severe disease, with a significant unmet need for approved treatment in patients with R/R EBV^+^ PTLD. Tabelecleucel was perceived as bringing improvements in terms of efficacy and safety in patients with R/R EBV^+^ PTLD compared to salvage therapy, the most representative comparators for tabelecleucel. Furthermore, in the discussion it was clarified that tumour exacerbation and pneumonitis were not related to tabelecleucel. Regarding the PROs and medical/non-medical costs associated with tabelecleucel, after the reflexive discussion, most experts considered that the high efficacy and safety results of tabelecleucel could represent an improvement in the quality of life of patients and savings compared to the salvage therapy. However, some experts still emphasized the need of more evidence to confirm these improvements and savings over time.

It is important to note that one expert shared during the reflective discussion his experience in clinical practice on the use of tabelecleucel for a paediatric patient with EBV^+^ PTLD, highlighting that the outcome was excellent. The paediatric patient who had previously undergone a multivisceral transplant, after more than 2 years of receiving tabelecleucel for R/R EBV^+^ PTLD, changed from being in palliative care to having a fully normal quality of life without immunosuppression and attending high school. This may have influenced the improvement of the tabelecleucel value contribution score during the re-test, so experience in clinical practice may also have influenced the assessment of the value contribution of the drugs. It is considered important that experts with experience in the drug participate in the reflective debate to resolve doubts and provide more data, creating an enriching discussion.

Regarding contextual criteria, the use of tabelecleucel to treat EBV^+^ PTLD was considered aligned with the priorities of NHS and has a positive impact on the specific interests of these population of patients and scientific societies. EBV^+^ PTLD was considered a very rare disease with no available alternatives, and tabelecleucel targets this unmet need. Experts agreed that the Spanish healthcare system would be prepared for the management of tabelecleucel in real practice as NHS has the capability and infrastructure required to ensure the proper use of tabelecleucel in treating EBV^+^ PTLD, especially centres with experience in CAR-T therapy which require more labour-intensive infrastructure.

This study shows that MCDA methodology would contribute to improve decision-making because it helps to consider the different stakeholder’s perspectives and enhance reflective discussion between them [33]. Health administration is complex and requires standardized, transparent, reflective, and value-based decision-making processes [34]. Therefore, it is understandable that MCDA is increasingly becoming popular for supporting healthcare decision-making [34].

MCDA is increasingly used to assess orphan drugs indicated for rare diseases [19, 21, 24, 35]. For example, Guarga et al. developed a framework adapted to the context of MCDA to assess orphan drugs in the Catalan Health System [17]. Similarly, the Working Group on Orphan Drugs and Rare Diseases of the Spanish Society of Hospital Pharmacy (Orphan-SEFH) uses the MCDA methodology for the elaboration of Orphan Drugs Assessment Reports [25].

The current analysis has some limitations. First, the study panel consisted of six experts; however, it is essential to highlight their extensive experience in the management of EBV^+^ PTLD and decision-making for ODs. In addition, the number of experts was in accordance with other MCDA studies [22, 35] and it was very similar to the number of experts that form evaluation commissions in Spain. Second, the study did not include any patients, so the patient perspective is not available. The EMA allows expert patients to bring their real-life experience of living with their condition directly into the scientific regulatory discussions [36]. However, EBV^+^ PTLD is a very rare disease so it would have been very difficult to find an expert patient to be involved in this study. Third, the results may depend to some extent on the composition of the expert panel, on their value judgements, experience, and training. To mitigate potential biases, all participants were trained in the MCDA methodology before scoring the evidence matrix.

This study supports that one of the most important steps in the standardized value assessment of innovative therapies is the reflective discussion enabled by the MCDA methodology. During the reflective discussion, all doubts of the participants who do not have experience in clinical practice with the evaluated drug could be resolved, so the value contribution of tabelecleucel obtained from the re-scoring, together with the justification of their scores, were much more reliable.

## Conclusions

The result of the study shows the importance of multidisciplinary reflective discussion for the assessment of the value contribution of a drug. Following the reflective discussion, an improvement in the standardised value contribution of tabelecleucel from 0.63 to 0.75 was reflected. Treatment with tabelecleucel has the potential to significantly improve the clinical course of EBV^+^ PTLD, a severe and life-threatening disorder. The MCDA methodology, due to its reflective and clarifying capacity, is proven to be an essential tool for decision-making on new treatments for the management of rare diseases, underlining the importance of thoughtful multidisciplinary discussion. The reflective discussion allows resolving possible doubts or misinterpretations of experts and the re-test enables obtaining a more consistent and reliable result on the value contribution of the drug, potentially more positive, and with less variation in the score.

## Data Availability

Due to participants privacy protection, the datasets used and/or analysed during the current study are available from the corresponding author upon reasonable request.

## Abbreviations

EBV^+^: Epstein Barr-virus-positive
EBV^+^ PTLD: Epstein-Barr virus-positive post-transplant lymphoproliferative disease
EMA: European medicines agency
HCT: Allogeneic haematopoietic cell transplant
MCDA: Multi-criteria decision analysis
NHS: National healthcare system
PROs: Patient-reported outcomes
PTLD: Post-transplant lymphoproliferative disease
R/R EBV^+^ PTLD: Refractory or relapsed epstein-barr virus-positive post-transplant lymphoproliferative disease
SOT: Solid organ transplant
